# Morphological characterization of *Calotropis procera* (Aiton) W.T. Aiton, a neglected medicinal plant

**DOI:** 10.1371/journal.pone.0341425

**Published:** 2026-02-10

**Authors:** Ali Khadivi, Farhad Mirheidari, Yazgan Tunç, Abdolvahid Saeidifar, Younes Moradi, Vali Rabiei, Farhang Razavi

**Affiliations:** 1 Department of Horticultural Sciences, Faculty of Agriculture and Natural Resources, Arak University, Arak, Iran; 2 Republic of Türkiye, Ministry of Agriculture and Forestry, General Directorate of Agricultural Research and Policies, Hatay Olive Research Institute Directorate, Hassa Station, Hassa, Hatay, Türkiye; 3 Ministry of Agriculture Jihad, Zahedan, Sistan-va-Baluchestan, Iran; 4 Department of Horticulture, Faculty of Agriculture, University of Zanjan, Zanjan, Iran; Central University of Punjab, INDIA

## Abstract

*Calotropis procera* (Aiton) W.T. Aiton, a medicinally significant but understudied species, exhibits remarkable adaptability to arid environments. Despite its ecological and pharmacological value, the species remains poorly characterized morphologically. This study evaluated the morphological diversity of 50 *C. procera* accessions from Sistan-va-Baluchestan province, Iran, using multivariate statistical approaches to identify key phenotypic variations and their potential applications. Significant morphological variability was observed among the accessions, with 37 out of 53 traits exhibiting coefficients of variation greater than 20%. Fruit-related traits, such as fruit length and weight, exhibited the highest variability, with the first principal component (PC1) explaining 17.45% of the total variance. Leaf characteristics, including color and pubescence, were dominant in the second principal component (PC2), accounting for 14.53% of the variance. Multiple regression analysis revealed a strong positive correlation between fruit weight and fruit length (*β* = 0.73, *p* ≤ 0.00). A trade-off was observed between flower number and fruit weight (*β* = −0.32, *p* ≤ 0.00). Heat map analysis grouped the accessions into four distinct clusters, with ‘Roodan-10’ and ‘Bahoukalat-1’ standing out for their superior fruit and floral traits, respectively. Notably, accessions such as ‘Dargas-10’ (100-seed weight: 2.40 g), ‘Rask-8’ (seed hair length: 30.19 mm), and ‘Pishin-8,’ distinguished by its exceptional leaf dimensions (mature leaf width: 139.20 mm), were identified as potential candidates for breeding. The remarkable leaf size of ‘Pishin-8’ suggests a high level of photosynthetic efficiency, which could enhance biomass production, further contributing to the ecological and agronomic value of this accession due to its exceptional seed characteristics. This study provides the first comprehensive morphological evaluation of *C. procera*, highlighting its high phenotypic diversity and adaptive traits. The findings underscore the species’ potential for medicinal and ecological applications, with specific accessions offering valuable genetic resources for conservation and breeding programs. Future research integrating molecular analyses is recommended to elucidate the genetic basis of observed variations.

## 1. Introduction

*Calotropis procera* (Aiton) W.T. Aiton, commonly known as the apple of Sodom, is a perennial shrub or small tree belonging to the Apocynaceae family [1]. This species is widely distributed across arid and semi-arid regions of Africa, the Middle East, the Indian subcontinent, and parts of South America [2]. It thrives in harsh environmental conditions, including saline soils, extreme drought, and high temperatures, highlighting its ecological adaptability and potential resilience in marginal lands [3]. Despite its widespread occurrence and ethnomedicinal significance, *C. procera* remains underutilized and neglected in scientific research and agricultural applications [4]. Understanding its morphological characteristics is crucial for conservation, genetic improvement, and potential commercial exploitation [5].

The medicinal value of *C. procera* is well established in traditional healthcare systems, including Ayurveda, Unani, and traditional African medicine [6,7]. Ethnomedicinal reports and pharmacological evidence indicate that multiple organs of the plant—particularly leaves, latex, flowers, and roots—exhibit diverse bioactivities such as analgesic, anti-inflammatory, antimicrobial, and anticancer effects [8,9]. Among these, the latex has attracted special attention because it is rich in bioactive constituents, notably cardenolides, flavonoids, alkaloids, and terpenoids, which collectively underpin its therapeutic potential [10,11]. Nevertheless, despite the recognized medicinal relevance of the species, research addressing intraspecific morphological diversity and trait variation among geographically distinct accessions remains limited [12]. A robust morphological characterization is therefore pivotal not only for documenting phenotypic diversity, but also for identifying superior accessions with desirable attributes that may support future medicinal utilization, ecological restoration, and industrial exploitation [13].

Morphological characterization is a fundamental step in plant taxonomy, conservation, and breeding programs. It provides essential insights into genetic diversity, adaptability, and phenotypic plasticity among accessions [14,15]. The study of morphological traits in *C. procera* can facilitate the identification of distinct morphotypes, which may be associated with specific environmental adaptations or therapeutic potential [3]. Additionally, clustering analysis based on morphological attributes can contribute to the classification of accessions into distinct groups, aiding in the selection of elite accessions for future research and cultivation [16].

Previous studies on *C. procera* have primarily focused on its phytochemical composition and pharmacological activities, while comprehensive morphological analyses remain scarce [17,18]. Given the increasing interest in sustainable and alternative medicinal resources, a systematic characterization of its morphological diversity is urgently needed. Such characterization not only provides a foundation for genetic improvement but also enhances the understanding of its ecological adaptations and potential applications in degraded environments.

This study aims to evaluate the morphological diversity of *C. procera* accessions using a systematic clustering approach. By analyzing a broad range of morphological traits, including leaf, flower, fruit, and seed characteristics, this research seeks to identify key phenotypic variations among accessions. The clustering patterns observed in this study will contribute to the taxonomic and agronomic understanding of *C. procera*, paving the way for its effective conservation and utilization in medicinal and ecological contexts.

## 2. Materials and methods

### 2.1. Plant material

**Fig 1:**
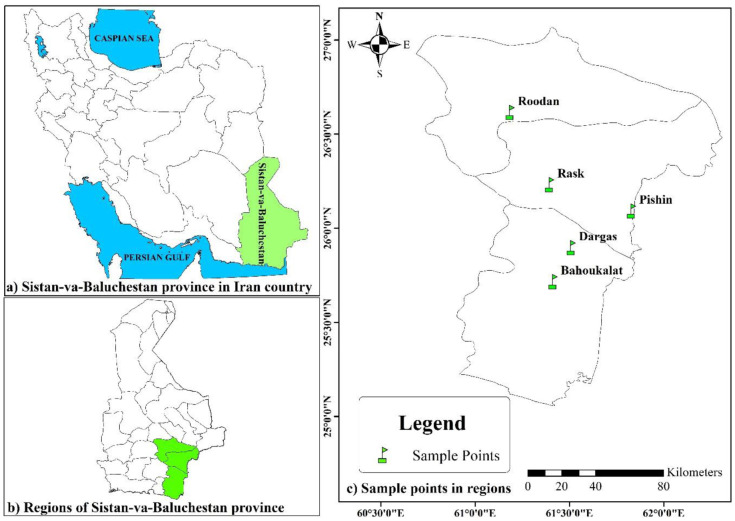
Geographic locations of collection sites of the studied *Calotropis procera* accessions. The map was generated by the first author, Ali Khadivi, using ArcGIS software v10.1 and is original; no third-party copyrighted or proprietary material was used, and no permission is required. (ArcGIS: http://www.arcgis.com/home/item.html?id=30e5fe3149c34df1ba922e6f5bbf808f).

Morphological variations of 50 natural accessions of *C. procera* were evaluated in five areas of Sistan-va-Baluchestan province, Iran, including Rask, Pishin, Roodan, Dargas, and Bahoukalat. The geographic locations of collection sites of the studied accessions are shown in Fig 1. The map was generated using ArcGIS software version 10.1 [19]. To ensure accurate sampling, a minimum distance of 200 m was maintained between accessions within each area, thereby preventing the collection of clone samples. Specimens were formally identified by Prof. Dr. Ali Khadivi. A herbarium voucher specimen with sediment number CP-4544 has been donated to the public available herbarium of the Faculty of Agriculture and Natural Resources of Arak University, Iran.

#### 2.1.1. Statement specifying permissions.

For this study, we acquired permission to study *C. procera* issued by the Agricultural and Natural Resources Ministry of Iran.

#### 2.1.2. Statement on experimental research and field studies on plants.

All sampled plants, whether cultivated or wild-growing, were collected in full accordance with institutional, national, and international guidelines, as well as the domestic legislation of Iran.

### 2.2. Morphological evaluations

Fifty-five morphological traits were used to evaluate phenotypic variation (Table 1). A total of 20 adult leaves and 20 mature fruits per accession were randomly selected and harvested. The traits related to the dimensions of leaf, fruit, and seed were measured using a digital caliper (Insize 1108−200). A digital scale with an accuracy of 0.01 g was used to measure the weight of fruit and seed (Swock JD602). The qualitative traits (Table 2) were visually examined and coded [20].

**Table 1:** Descriptive statistics for the morphological traits utilized in the studied *Calotropis procera* accessions.

Trait	Abb.	Unit	Min.	Max.	Mean	±SD	CV (%)
Plant growth habit	V1	Code	1	7	3.32	2.15	64.82
Plant growth vigor	V2	Code	1	5	3.32	1.24	37.23
Plant height	V3	Code	1	5	2.96	1.25	42.06
Plant breadth	V4	Code	1	5	3.00	1.46	48.57
Canopy density	V5	Code	1	5	2.84	1.45	50.99
Plant branching	V6	Code	1	5	2.84	1.50	52.92
Stem diameter	V7	Code	1	5	2.68	1.24	46.12
Main stem bark cracks	V8	Code	1	5	1.88	1.41	74.95
Leaf density	V9	Code	1	5	3.92	1.09	27.68
Young leaf shape	V10	Code	1	5	2.28	1.60	70.35
Young leaf length	V11	mm	34.77	92.86	61.60	14.35	23.30
Young leaf width	V12	mm	16.70	54.09	31.75	9.16	28.84
Young leaf apex shape	V13	Code	1	3	2.52	0.86	34.25
Young leaf base shape	V14	Code	1	5	3.92	1.47	37.47
Young leaf color	V15	Code	1	5	3.24	1.49	46.05
Young leaf vein color	V16	Code	1	3	1.96	1.01	51.48
Young leaf pubescence	V17	Code	1	5	3.24	1.49	46.05
Young leaf thickness	V18	mm	0.36	0.95	0.57	0.14	24.01
Mature leaf shape	V19	Code	1	5	2.28	1.13	49.39
Mature leaf length	V20	mm	74.83	188.22	126.45	25.98	20.54
Mature leaf width	V21	mm	37.24	139.20	76.55	18.90	24.69
Mature leaf apex shape	V22	Code	1	3	2.72	0.70	25.77
Mature leaf color	V23	Code	1	5	3.32	1.42	42.80
Mature leaf vein color	V24	Code	1	3	1.84	1.00	54.18
Mature leaf pubescence	V25	Code	1	5	1.72	1.13	65.47
Mature leaf thickness	V26	mm	0.51	1.30	0.81	0.19	23.81
Inflorescence diameter	V27	mm	54.20	95.60	73.42	11.85	16.15
Number of flowers in inflorescence	V28	Number	10	53	27.40	11.14	40.65
Peduncle length	V29	mm	41.27	105.65	72.79	16.02	22.01
Peduncle diameter	V30	mm	4.18	9.03	5.77	1.01	17.44
Pedicel length	V31	mm	6.95	27.32	16.86	3.59	21.30
Pedicel diameter	V32	mm	0.95	2.41	1.53	0.30	19.33
Flower diameter	V33	mm	18.40	30.10	25.73	2.38	9.26
Petal length	V34	mm	9.43	15.25	12.28	1.47	11.96
Petal width	V35	mm	5.22	9.17	7.22	0.92	12.74
Petal color	V36	Code	1	5	3.12	1.53	49.17
Corona lobe length	V37	mm	2.00	3.09	2.60	0.28	10.58
Stigmatic disc width	V38	mm	2.65	5.14	4.64	0.35	7.49
Sepal length	V39	mm	4.13	6.87	5.45	0.73	13.45
Sepal width	V40	mm	2.35	4.30	3.32	0.40	12.04
Fruit density	V41	Code	1	5	3.72	1.39	37.26
Fruit length	V42	mm	52.65	91.20	69.57	9.43	13.55
Fruit width	V43	mm	22.30	49.70	32.27	7.52	23.32
Fruit thickness	V44	mm	21.22	51.35	32.07	6.90	21.52
Fruit stalk length	V45	mm	16.83	68.98	26.77	7.68	28.68
Fruit stalk diameter	V46	mm	1.99	3.98	3.44	0.44	12.75
Fruit weight	V47	g	6.85	26.70	15.81	4.36	27.56
Fruit skin color	V48	Code	1	3	2.08	1.01	48.41
Seed length	V49	mm	5.62	8.67	7.13	0.96	13.42
Seed width	V50	mm	3.66	5.20	4.33	0.38	8.76
Seed thickness	V51	mm	0.75	1.32	1.03	0.14	13.64
100-seed weight	V52	g	0.90	2.40	1.44	0.34	23.25
Seed hair length (fibers)	V53	mm	13.20	30.19	22.79	3.94	17.30

*Abb* Abbreviations, *Max* Maximum, *Min* Minimum, *± SD* Standard Deviation, *CV* Coefficient of Variation.

**Table 2:** Frequency distribution for the measured qualitative morphological characters in the studied *Calotropis procera* accessions.

Trait	Frequency (No. of accessions)			
	1	3	5	7
Plant growth habit	Prostrate (18)	Open (13)	Semi-erect (12)	Erect (7)
Plant growth vigor	Low (6)	Moderate (30)	High (14)	–
Plant height	Low (10)	Moderate (31)	High (9)	–
Plant breadth	Low (13)	Moderate (24)	High (13)	–
Canopy density	Low (15)	Moderate (24)	High (11)	–
Plant branching	Multi-stem/Low (16)	Multi/Intermediate (22)	Multi/High (12)	–
Stem diameter	Low (14)	Moderate (30)	High (6)	–
Main stem bark cracks	Low (34)	Moderate (10)	High (6)	–
Leaf density	Low (1)	Moderate (25)	High (24)	–
Young leaf shape	Ovate (28)	Oval (12)	Obovate (10)	–
Young leaf apex shape	Acute (12)	Acuminate (38)	–	–
Young leaf base shape	Rounded (7)	Truncate (13)	Cordate (30)	–
Young leaf color	Light green (11)	Green (22)	Dark green (17)	–
Young leaf vein color	Cream (26)	Cream-green (24)	–	–
Young leaf pubescence	Low (11)	Moderate (22)	High (17)	–
Mature leaf shape	Ovate (20)	Oval (28)	Obovate (2)	–
Mature leaf apex shape	Acute (7)	Acuminate (43)	–	–
Mature leaf color	Light green (9)	Green (24)	Dark green (17)	–
Mature leaf vein color	Cream (29)	Cream-green (21)	–	–
Mature leaf pubescence	Low (34)	Moderate (14)	High (2)	–
Petal color	White-light purple (13)	White-purple (21)	White-dark purple (16)	–
Fruit density	Low (6)	Moderate (20)	High (24)	–
Fruit skin color	Light green (23)	Green (27)	–	–

### 2.3. Statistical analysis

Analysis of variance (One-way ANOVA, *p < 0.05*) was performed to evaluate the variation among accessions based on the traits measured using JMP^®^ Pro 17 software [21]. Pearson (*r*) correlation coefficients were used to determine the relationship between the recorded traits using Origin Pro^®^ 2025b software [22]. Principal component analysis (PCA) was applied using Origin Pro^®^ 2025b software to identify the key traits influencing accession grouping. To enhance the interpretability of the components, the Varimax rotation with the Kaiser Normalization method was employed. This approach clarified the relationships between components, ensuring a more comprehensible and meaningful analysis. Heat map analysis based on Ward’s method and Euclidean distance coefficients using Origin Pro^®^ 2025b software was used to classify accessions and variables. The first and second principal components (PC1/PC2) were used to draw a two-dimensional biplot by determining the distribution of accessions and quantitative variables using Origin Pro^®^ 2025b software. In addition, a region-based PCA was applied to evaluate inter-regional relationships, and population-level differentiation was examined in the PC1–PC2 ordination space. Moreover, fruit-related traits were considered dependent variables, and the characteristics affecting these characters were determined using multiple regression analysis (MRA). The MRA was conducted using the “stepwise” method of the “linear regression analysis” option of SPSS^®^ (SPSS Inc., Chicago, IL, USA) [23,24]. Multivariate approaches (e.g., PCA and hierarchical clustering) were used to summarize trait patterns and to support accession discrimination, consistent with recent morpho-biochemical diversity studies in underutilized crops [25–29].

## 3. Results and discussion

### 3.1. Descriptive statistics among accessions

Descriptive statistics for the morphological traits utilized in the studied *C. procera* accessions are presented in Table 1. Previous studies [20,30–34] identified that certain variables exhibited no significant variation among accessions (CV = 0.00%), prompting their exclusion from further analysis. In line with these findings, two variables (mature leaf base shape and seed shape) were found to lack significant variation (CV = 0.00%) among the accessions in this study. Consequently, the evaluations were conducted based on the remaining 53 variables. In this regard, the one-way ANOVA (*p < 0.05*) demonstrated significant differences among the evaluated accessions. The highest variation was observed in main stem bark cracks (74.95%), young leaf shape (70.35%), mature leaf pubescence (65.47%), plant growth habit (64.82%), and mature leaf vein color (54.18%). In contrast, the lowest variation was recorded in petal length (11.96%), corona lobe length (10.58%), flower diameter (9.26%), seed width (8.76%), and stigmatic disc width (7.49%). Notably, 37 out of 53 variables (representing 69.81% in total) had coefficients of variation (CVs) greater than 20.00%. This value indicates a significant level of variability among the studied accessions [35]. Traits with a coefficient of variation (CV) greater than 20.00% are more distinguishable among specimens and can serve as reliable markers for differentiating accessions, genotypes, or cultivars [36]. Additionally, traits with a broader quantitative range generally show higher CV% values, reflecting greater potential for selection and improvement [37]. On the other hand, morphological traits with lower CV values are more consistent and can be regarded as stable characteristics across accessions [36].

The CV values reported in similar studies on different species indicate that genetic diversity may vary across species. For instance, CV values of 68.19% for *Elaeagnus angustifolia* [38], 62.50% for cornelian cherry [39], and 70.00% for pomegranate (*Punica granatum*) [40] have been documented. These high CV values suggest substantial phenotypic variability among the studied accessions.

Overall, the variability observed across the morphological traits in *C. procera* accessions aligns with previous studies on other species, where high CVs indicate significant phenotypic diversity [38,40,39]. This degree of morphological heterogeneity suggests that these accessions may possess valuable genetic resources for future breeding programs. The observed variation also emphasizes the importance of morphological characterization in understanding the adaptive potential and agronomic value of underutilized medicinal plants like *C. procera*.

The measurements of leaf traits demonstrate a broad range, suggesting notable differences among the accessions. Young leaf length varies between 34.77 (‘Rask-6’) and 92.86 mm (‘Roodan-2’), while mature leaf length ranges from 74.83 (‘Rask-7’) to 188.22 mm (‘Pishin-1’). This high level of variation may reflect both genetic and environmental influences, which are crucial factors for identifying superior accessions with improved leaf biomass or other desirable characteristics. Furthermore, the differences in leaf thickness, ranging from 0.36 (‘Dargas-5’) to 0.95 mm (‘Bahoukalat-1’) in young leaves and 0.51 (‘Pishin-2’) to 1.30 mm (‘Bahoukalat-2’) in mature leaves, highlight additional morphological diversity within the population. Likewise, leaf width varied widely among accessions: young leaves ranged from 16.70 mm in ‘Dargas-11’ to 54.09 mm in ‘Roodan-2’, whereas mature leaves ranged from 37.24 mm in ‘Rask-8’ to 139.20 mm in ‘Pishin-8’.

Floral traits also exhibit substantial variability. The number of flowers per inflorescence ranges from 10 (‘Dargas-2’) to 53 (‘Bahoukalat-4’), indicating considerable differences in reproductive output across accessions. This variation may be valuable for breeding programs focusing on enhancing flower production or other floral characteristics. The inflorescence diameter shows a range of 54.20 (‘Bahoukalat-8’) to 95.60 mm (‘Bahoukalat-1’), which, combined with differences in peduncle and pedicel dimensions, suggests a broad morphological diversity in floral structures. Such variability is essential for understanding the reproductive biology of *C. procera* and may provide insight into traits associated with pollination efficiency or seed production.

Fruit-related traits also display extensive variation, with fruit length ranging from 52.65 mm to 91.20 mm and fruit width between 22.30 (‘Bahoukalat-8’) and 49.70 mm (‘Roodan-10’). The observed differences in fruit thickness [21.22 (‘Rask-8’) to 51.35 mm (‘Roodan-10’)] and fruit weight [6.85 (‘Rask-6’) to 26.70 g (‘Roodan-6’)] suggest that certain accessions may possess superior fruit yield potential. Additionally, the variability in fruit stalk length and diameter reflects morphological differences that could influence mechanical harvesting or fruit handling. These findings indicate that specific accessions may be selected for their superior fruit size and mass, contributing to breeding programs focused on increasing yield and improving fruit quality.

Seed traits exhibit a more moderate but still notable degree of variation. Seed length ranges from 5.62 (‘Dargas-6’) to 8.67 mm (‘Pishin-10’), while seed width varies between 3.66 (‘Bahoukalat-2’) and 5.20 mm (‘Roodan-8’). The thickness of the seeds, which ranges from 0.75 (‘Bahoukalat-4’) to 1.32 mm (‘Rask-2’), suggests structural differences that could impact germination rates or seed viability. The 100-seed weight demonstrates variation from 0.90 (‘Bahoukalat-1’) to 2.40 g (‘Dargas-10’), which is an important indicator of seed quality and potential vigor. Additionally, the length of seed hairs (fibers) ranges from 13.20 (‘Bahoukalat-1’) to 30.19 mm (‘Rask-8’), representing a substantial range that may influence seed dispersal mechanisms.

The leaves of *C. procera* are simple, sub-sessile, and arranged in an opposite decussate pattern. They exhibit a somewhat thick or fleshy texture and vary in shape from oblong and obovate to nearly orbicular. The apex is short-pointed to blunt, while the base is heart-shaped and clasping. The leaf blades display a color spectrum from light to dark green, with nearly white venation. Initially pubescent, the leaves become glabrous on both surfaces upon maturity. A fine layer of soft hairs, which can be easily removed, covers the surface, contributing to their slightly leathery texture. Additionally, the leaves possess a waxy appearance and exude white milky latex when damaged [41,42]. They are relatively large, reaching up to 15 cm in length and 10 cm in width, and lack a distinct petiole [43].

Flowering occurs continuously throughout the year [44]. The flowers are actinomorphic and bisexual, and emit a subtle fragrance. They develop in clusters of ovoid buds positioned at the axils of the uppermost leaves, forming axillary inflorescences. Each cluster comprises between 3 and 15 flowers, enclosed by an involucre of multiple small, oblong, pointed, scaly, and caducous bracts. The primary stalk of these inflorescences (peduncle) measures between 20 and 55 mm in length, while individual flowers are supported by pedicels ranging from 15 to 25 mm. Both the calyx and corolla exhibit five lobes; the sepals, which are ovate and acute, are approximately 7–8 mm long and covered with fine hairs externally. The petals, measuring 2–3 cm in width, are white with purple tips on the inner surface. The fruit is inflated, ovoid in shape, and reaches a length of 8–12 cm [45]. Additionally, the flowers feature a distinctive crown-like structure known as the corona [46,43]. The androecium comprises five stamens that are inserted at the base of the corolla. These five staminal structures are adnate and fused with the gynoecium, resulting in the formation of a gynostegium. The filaments merge to create a prominent staminal column, which bears five distinct, radiating coronal appendages. These appendages are entirely adnate to the column but are slightly shorter in length. The anthers are relatively short, broad, and somewhat rigid, with membranous, broadly triangular anther tips that bend inward, covering the sides of the stigmatic hood. Each flower secretes a substantial amount of nectar [47]. The gynoecium of *C. procera* consists of two separate carpels that are fused at the apex of the style. The stigma is broad and pentagonal, with the actual stigmatic surfaces located within stigmatic chambers beneath the stigmatic lobes, positioned between adjacent anthers. The corona consists of five fleshy, laterally compressed, purple lobes extending outward from the gynostegium. Each lobe features a recurved vesicle at its base and a bifurcated apex with an external cleft [48,46].

The fruits of *C. procera* are follicles measuring 8–14 cm in length and 6–9 cm in width, exhibiting a shape that varies from sub-globose to obliquely ovoid. Their apex is rounded, with a green, spongy, and smooth surface. Upon reaching maturity, the fruits split open and invert to facilitate seed dispersal [49,42]. Each fruit contains a large number of seeds, ranging from 350 to 500. The seeds are flat, obovate in shape, and approximately 6 × 5 mm in size. They are equipped with a silky white pappus that extends 3 cm or more, aiding in wind dispersal [50]. Fruiting occurs throughout the year [44].

The morphological measurements obtained in our study show both similarities and differences when compared to the findings of previous studies. The observed differences may vary depending on factors such as genetic variation, ecological conditions, microclimatic factors in the growing environment, and environmental stress conditions. Identifying these differences plays a crucial role in understanding the phenotypic flexibility and environmental adaptation capacity of the species. Moreover, determining the variation among individuals growing in different ecologies provides valuable insights for the conservation of genetic resources and the selection of superior accessions. In this regard, a detailed analysis of morphological variability will contribute to a better understanding of the species' taxonomic classification and its potential applications.

Notably, fiber-related traits in *C. procera* have recently been investigated at the genomic level; a genome-wide association study (GWAS) identified significant SNP markers and candidate genomic regions linked with fruit fiber traits (including fiber length), highlighting a genetic basis for fiber variation and supporting the integration of phenotypic screening with molecular evidence in future breeding efforts [51].

Frequency Distribution is a statistical method in which the elements of a dataset are grouped into specific categories or ranges, and the number of elements (frequencies) within each group is determined. This distribution allows for understanding how the data is distributed and which categories or ranges occur more or less frequently. Frequency distribution is a fundamental tool in data analysis, particularly when it comes to the quantification of qualitative data [52]. In this study, numerical values for ordinal data were used for the analyses.

**Fig 2:**
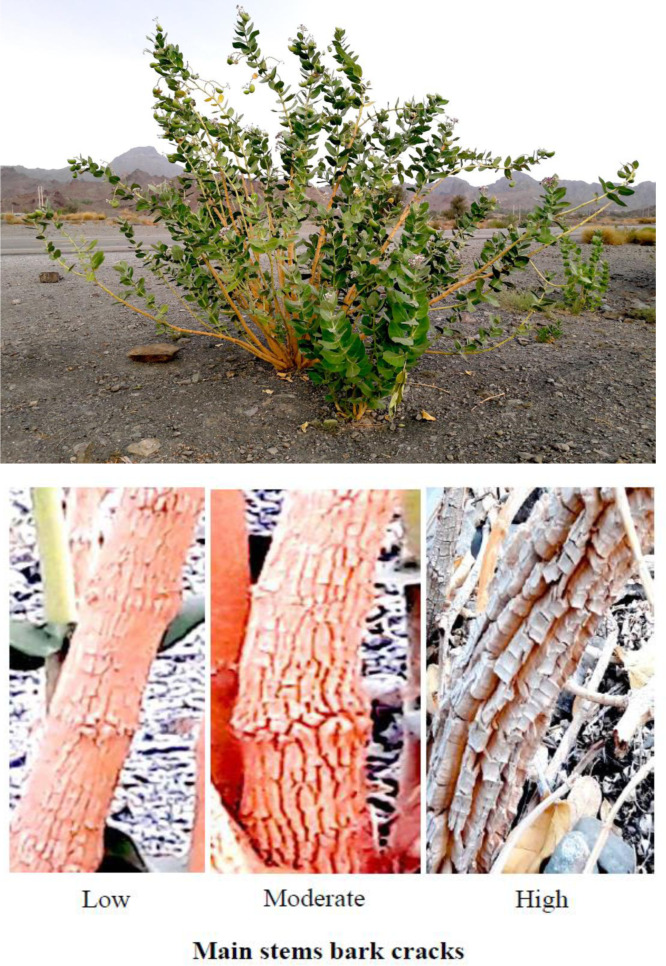
The shrub and stem of the studied *Calotropis procera* accessions. These images were taken by the second author, Farhad Mirheidari.

**Fig 3:**
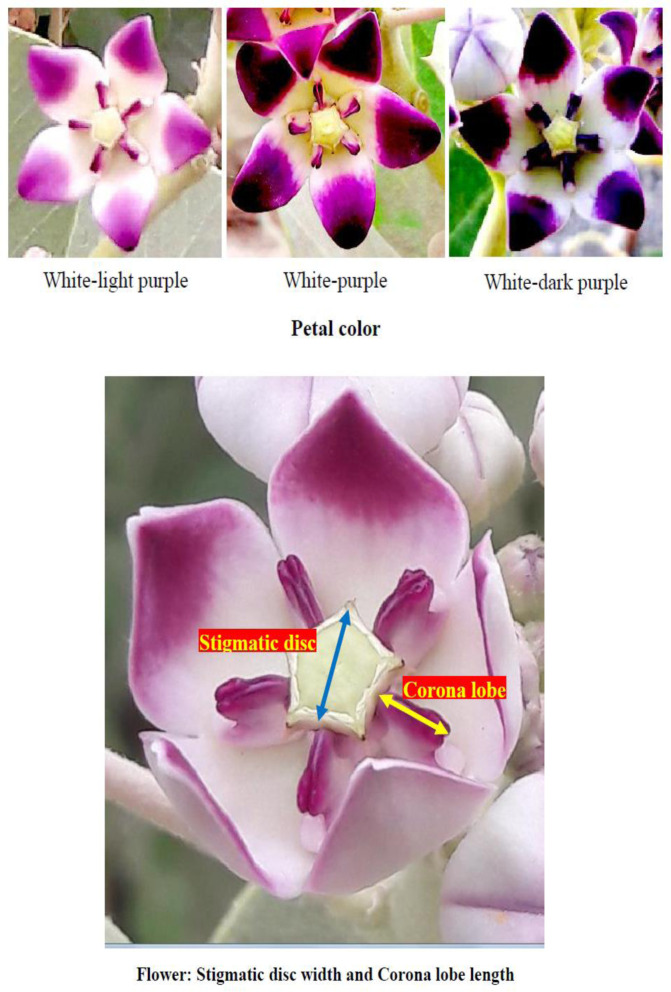
The flower of the studied *Calotropis procera* accessions. These images were taken by the second author, Farhad Mirheidari.

**Fig 4:**
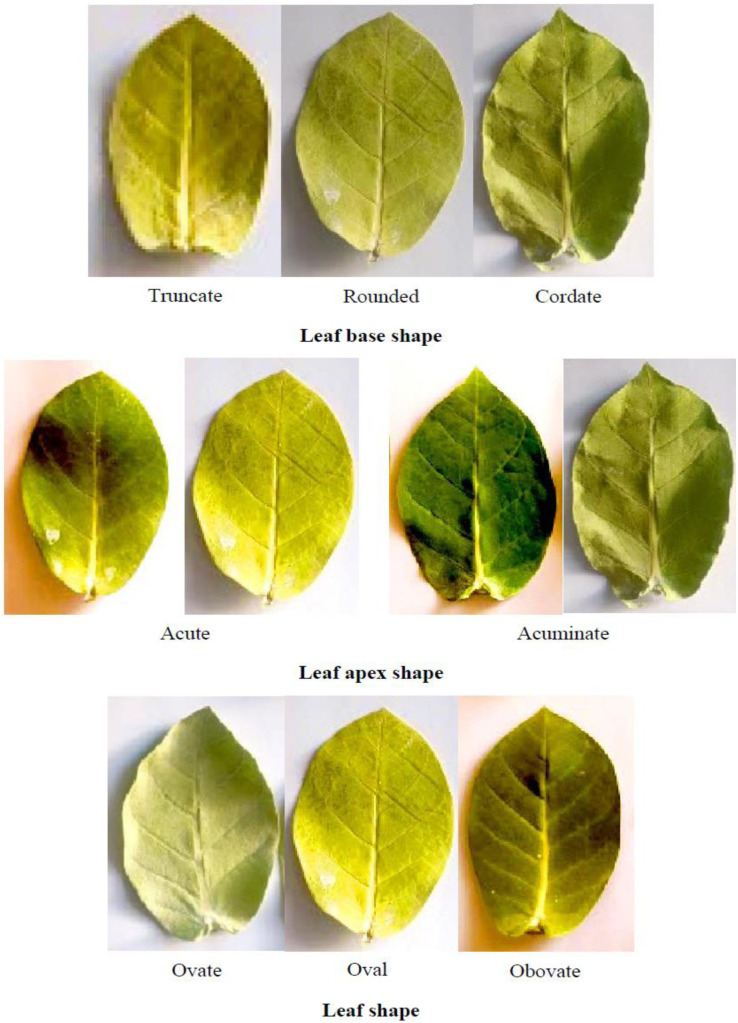
The leaf of the studied *Calotropis procera* accessions. These images were taken by the second author, Farhad Mirheidari.

**Fig 5:**
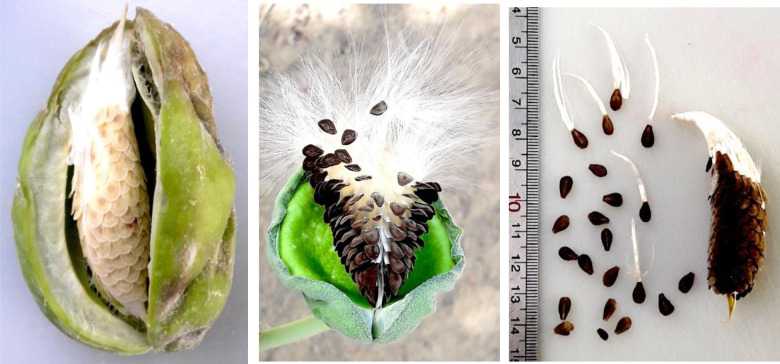
The fruit and seed of the studied *Calotropis procera* accessions. These images were taken by the second author, Farhad Mirheidari.

The frequency distribution table presents absolute counts of morphological traits observed across 50 *C. procera* accessions, revealing distinct patterns in this medicinal species (Table 2). The growth habit data indicate prostrate forms were most common (18 accessions), followed by open (13), semi-erect (12), and erect (7) types, indicating a predominance of spreading growth architectures. Growth vigor displays a similar trend, with moderate vigor appearing in 30 accessions compared to just 6 showing low vigor and 14 with high vigor, suggesting generally robust growth across the population. Structural characteristics exhibit consistent patterns, where moderate expressions predominate for plant height (31 accessions), breadth (24), and canopy density (24). Stem diameter suggests a particularly skewed distribution, with 30 accessions falling in the moderate range versus only 6 showing high values, potentially indicating environmental or developmental constraints on stem thickening. Bark cracking appears limited in most accessions (34 showing low cracking), possibly reflecting a conserved protective mechanism. Leaf morphology presents particularly notable variations. Young leaf shape shows ovate forms as most frequent (28 accessions), with oval (12) and obovate (10) less common. The apex shape strongly favors acuminate types (38 accessions) over acute (12), while cordate bases dominate (30 accessions) compared to rounded (7) or truncate (13) forms. Leaf coloration trends toward intermediate green tones (22 accessions for young leaves, 24 for mature), with dark green forms well-represented (17 accessions for both leaf ages). Pubescence decreases dramatically with leaf maturity, dropping from 17 accessions showing high pubescence in young leaves to just 2 in mature leaves. Reproductive structures display balanced distributions, with petal coloration showing white-purple (21 accessions) slightly more common than white-dark purple (16) or white-light purple (13). Fruit characteristics show high density in 24 accessions versus low density in just 6, with skin color predominantly green (27 accessions) over light green (23). These absolute counts provide a foundation for understanding trait variability in this species, where certain characteristics like acuminate leaf apices appear strongly conserved, while others like growth habits show broader variation across accessions. The data suggest both developmental constraints and potential adaptive significance for particular morphological features in *C. procera*. The shrub, stem, flower, leaf, fruit, and seed of the studied *C. procera* accessions are shown in Figs 2–5.

### 3.2. Correlation matrix analysis (CMA)

Correlation matrix analysis is a statistical method used to examine the linear relationships between multiple quantitative variables simultaneously [53]. This approach is particularly useful in identifying the degree and direction of associations within a dataset. Each value within the matrix represents a correlation coefficient that quantifies the strength of the relationship between two variables. When analyzing quantitative data, the Pearson correlation coefficient is the most commonly used measure [54]. It calculates the linear relationship between variables and provides a value ranging from −1 to +1. A value closer to +1 indicates a strong positive relationship, meaning that as one variable increases, the other increases as well. Conversely, a value closer to −1 signifies a strong negative relationship, where an increase in one variable corresponds to a decrease in the other. A value around 0 suggests no linear association between the variables [55].

The Pearson correlation coefficient assumes that the data are normally distributed and that the relationship between variables is homoscedastic. This method is susceptible to outliers, which can significantly affect the results [56]. In research involving quantitative variables, correlation matrix analysis is frequently used to detect relationships, assess multicollinearity before applying advanced statistical techniques, and interpret the structure of complex datasets [57]. By systematically examining pairwise correlations, this analysis provides a clear understanding of how variables interact, which is crucial for informed decision-making and further statistical modeling.

**Fig 6:**
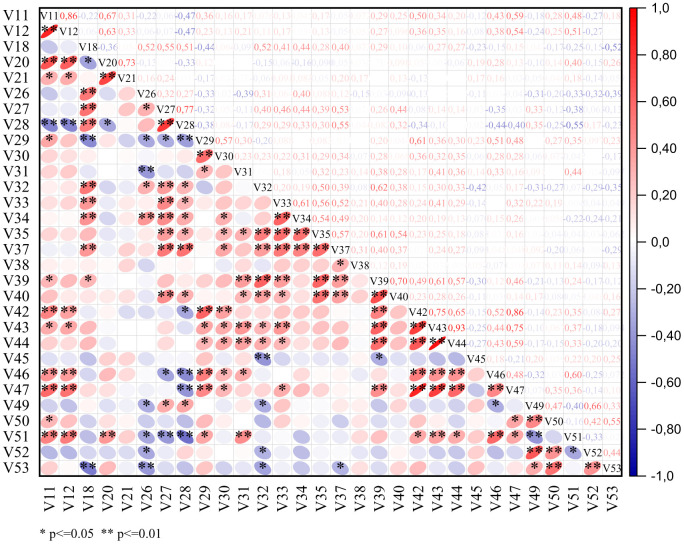
Simple correlations between the quantitative morphological variables utilized in the studied *Calotropis procera* accessions. For abbreviations, please see Table 1.

The correlation analysis of morphological traits in *C. procera* provides valuable insights into the interrelationships among key vegetative and reproductive parameters (Fig 6 and S1 Table). Fruit weight exhibited significant positive correlations with young leaf length (*r* = 0.59**) and width (*r* = 0.54**), suggesting that larger leaves during the juvenile stage may contribute to enhanced photosynthetic activity, ultimately supporting fruit development. Similarly, its positive correlations with fruit length (*r* = 0.86**), fruit width (*r* = 0.75**), and fruit thickness (*r* = 0.59**) indicate that heavier fruits tend to be larger in multiple dimensions, a pattern frequently observed in fleshy-fruited plant species. Among floral traits, fruit weight showed a moderate positive correlation with peduncle length (*r* = 0.48**) and diameter (*r* = 0.28*), implying that a more robust floral structure might facilitate better fruit set and development. Moreover, flower diameter (*r* = 0.32*) and sepal length (*r* = 0.46**) were also positively associated with fruit weight, reflecting the potential influence of larger floral organs on subsequent fruit formation. Conversely, fruit weight displayed a significant negative correlation with the number of flowers per inflorescence (*r* = −0.40**), indicating a possible trade-off between flower production and fruit development. Plants investing in a higher number of flowers may allocate fewer resources per fruit, potentially leading to reduced fruit size and weight. Additionally, the positive associations between fruit weight and fruit stalk diameter (*r* = 0.48**), seed width (*r* = 0.35*), and seed thickness (*r* = 0.36*) suggest that heavier fruits tend to produce larger seeds, which could enhance seedling vigor and establishment.

The 100-seed weight parameter exhibited notable relationships with leaf, floral, and seed traits. Its significant negative correlation with mature leaf thickness (*r* = −0.32*) might indicate a resource allocation trade-off, where plants producing heavier seeds invest less in leaf structural robustness. Among floral traits, a negative correlation was observed with pedicel diameter (*r* = −0.29*), suggesting that a reduction in floral support structures might be linked to increased seed mass. Regarding seed characteristics, 100-seed weight showed a strong positive correlation with seed length (*r* = 0.66**) and a moderate correlation with seed width (*r* = 0.42**), supporting the general pattern that heavier seeds tend to be larger. However, its negative correlation with seed thickness (*r* = −0.33*) indicates that while heavier seeds are elongated, they may not necessarily be proportionally thicker. Additionally, the significant positive association between 100-seed weight and seed hair length (*r* = 0.44**) suggests that heavier seeds might be equipped with longer fibers, potentially enhancing dispersal efficiency via wind.

These findings highlight important developmental trade-offs and potential functional implications in *C. procera*. The observed correlations suggest that leaf and floral traits play a critical role in determining fruit and seed attributes, emphasizing the plant’s resource allocation strategies. The negative association between flower number and fruit weight underscores a reproductive trade-off, while the relationships among seed traits indicate distinct strategies for optimizing dispersal and germination. Understanding these interdependencies can contribute to conservation efforts and potential agricultural utilization of this neglected medicinal species.

CMA has been conducted on *C. procera* in Benin [2] and Pakistan [58]. However, since previous studies did not include morphological variables, the findings obtained in this study were evaluated independently of each other. This approach allows for a more detailed examination of the intrinsic relationships among morphological variables and highlights the analytical originality of our study.

### 3.3. Multiple regression analysis (MRA)

Multiple regression analysis (MRA) is a statistical method used to examine the relationship between a dependent variable and multiple independent variables [59]. This analysis determines the effects of independent variables on the dependent variable and evaluates the contribution of each variable separately. The stepwise method employed in multiple regression analysis is an approach for selecting variables that have a significant effect on the dependent variable [60]. Several key statistical measures are evaluated in this analysis. The *r* correlation coefficient indicates the direction and strength of the linear relationship between the dependent variable and the independent variable(s), ranging from −1 to +1. The *r²* coefficient of determination represents the proportion of the total variance in the dependent variable explained by the independent variables and reflects the explanatory power of the model. A higher *r²* value indicates that the model explains the dependent variable more effectively. The *β* standardized beta coefficients measure the relative effects of the independent variables on the dependent variable, allowing for direct comparison by eliminating unit differences between variables. As the absolute value of the beta coefficient increases, the influence of that variable on the dependent variable becomes stronger [61]. The stepwise method is useful because it provides an automated process for variable selection, focusing on variables that meet the significance threshold (*p* ≤ 0.05) [62].

The results of the MRA reveal significant morphological predictors of fruit and seed weight in *C. procera* (Table 3). Fruit weight is most strongly influenced by fruit length, as indicated by the high standardized beta coefficient (***β* = 0.73**, ***p* ≤ 0.00**), suggesting that longer fruits tend to be heavier. This relationship is likely due to increased resource allocation and structural development in elongated fruits. Additionally, young leaf width (***β* = 0.19**, ***p* ≤ 0.00**) and thickness (*β* = 0.37, *p* ≤ 0.00) show positive associations with fruit weight, implying that larger and thicker young leaves may enhance photosynthetic capacity or nutrient supply during fruit development. Interestingly, the number of flowers in an inflorescence has a negative effect (***β* = −0.32**, ***p* ≤ 0.00**), possibly reflecting a trade-off between flower quantity and resource investment in individual fruits. There is a statistically significant, yet relatively weak, positive relationship between seed length and fruit weight (*β* = 0.18, *p* < 0.00). This result suggests that longer seeds may slightly increase fruit weight. However, the low value of *β* (0.18) indicates that this relationship has a secondary effect on fruit weight, and other factors (such as fruit length or leaf morphology) likely play a more dominant role.

**Table 3:** The characteristics associated with fruit and seed weight in the studied *Calotropis procera* accessions, as revealed using MRA and coefficients.

Dependant character	Independent character	*r*	*r* ^2^	*β*	*t* value	*p* value
Fruit weight	Fruit length	0.858^a^	0.74	**0.73**	14.05	0.00*
	Young leaf width	0.893^b^	0.80	**0.19**	3.35	0.00*
	Young leaf thickness	0.919^c^	0.84	0.37	6.41	0.00
	Number of flowers in inflorescence	0.936^d^	0.88	**−0.32**	−4.66	0.00*
	Seed length	0.950^e^	0.90	0.18	3.46	0.00
100-seed weight	Seed length	0.657^a^	0.43	**0.59**	**5.33**	0.00*
	Inflorescence diameter	0.720^b^	0.52	−0.57	−3.98	0.00
	Number of flowers in inflorescence	0.751^c^	0.56	0.46	3.02	0.00
	Seed hair length (fibers)	0.787^d^	0.62	**0.27**	**2.58**	0.01*

*r* Correlation coefficient, *r²* Coefficient of determination, *β* Standardized beta coefficients.

*Multiple regression analysis correlations supported by the correlation matrix analysis.

For 100-seed weight, seed length emerges as the most influential factor (***β* = 0.59**, ***p* ≤ 0.00**), consistent with the expectation that longer seeds contain more storage tissues. In contrast, inflorescence diameter exhibits a negative relationship (*β* = −0.57, *p* ≤ 0.00), which could indicate that larger inflorescences distribute resources more thinly among seeds, reducing individual seed mass. The positive contribution of the number of flowers in an inflorescence (*β* = 0.46, *p* ≤ 0.00) suggests that while higher flower numbers may reduce fruit weight, they could promote seed development through increased pollination efficiency or compensatory resource allocation. Lastly, seed hair length (***β* = 0.27**, ***p* ≤ 0.01**) shows a modest positive correlation, possibly linked to dispersal efficiency or seed coat development. The bold values are supported by the correlation matrix analysis.

These findings underscore the complex interplay between morphological traits and reproductive output in *C. procera*. The contrasting effects of certain traits, such as inflorescence size on fruit versus seed weight, highlight potential trade-offs in resource allocation. Further studies could explore the physiological mechanisms underlying these relationships, such as nutrient partitioning or hormonal regulation, to better understand the species’ adaptive strategies in arid environments.

Our research demonstrates that multiple regression analysis has been applied for the first time to the species *C. procera* in this study. Consequently, the obtained findings could not be directly compared with previous studies and were evaluated independently of each other. This not only highlights the novelty of our research but also establishes a foundation for future comparative analyses. The results provide valuable insights into the relationship between the morphological characteristics of the species and environmental factors, contributing to a deeper understanding of its phenotypic responses and ecological adaptability.

### 3.4. Principal component analysis (PCA)

Principal component analysis (PCA) is a statistical method used to reduce high-dimensional datasets to a lower-dimensional plane for more efficient analysis. PCA derives independent components, known as principal components, which are linear combinations of correlated variables, to better understand the underlying structures and relationships within the data. These components are ordered to explain the maximum variance in the original dataset [63]. The extraction method is employed to identify the principal components of the dataset, with each component being ordered to explain the greatest variance. The output of PCA is typically interpreted based on eigenvalues. In this context, only components with an eigenvalue ≥ 1.00 are considered, as these components represent a significant portion of the explained variance, while components with lower eigenvalues do not contribute meaningfully to the model’s explanatory power [64]. The transformation method involves Varimax rotation, a type of orthogonal rotation applied to enhance the interpretability of the components [65]. This method simplifies the relationships between components, ensuring that each component strongly represents a specific set of variables [66]. Varimax rotation, combined with Kaiser Normalization, also balances the amount of explained variance across components, thereby providing a more homogenous explanation [67]. PCA can be integrated with both quantitative and qualitative data. This integration allows for the simultaneous analysis of quantitative and qualitative data in complex datasets, making PCA a more flexible and powerful analytical tool [68]. In this study, PCA, Varimax rotation, Kaiser Normalization, and the eigenvalue ≥ 1.00 criterion were applied, with the first 15 principal components being considered (Table 4). These 15 components accounted for 85.60% of the total variance. Statistically, it was found that PC1 through PC7 were significant at *p* < 0.01, while PC8 through PC14 were significant at *p* < 0.05. PC15, however, was not significant.

**Table 4:** Eigenvalues of the principal component axes from the PCA of morphological characters in the studied *Calotropis procera* accessions.

Trait	Component														
	1	2	3	4	5	6	7	8	9	10	11	12	13	14	15
Plant growth habit	−0.05	−0.01	−0.32	−0.16	0.08	−0.13	0.19	−0.02	0.01	0.24	0.06	−0.01	−0.17	**0.74** ^**a**^	−0.12
Plant growth vigor	0.05	−0.12	0.59	−0.03	0.23	−0.10	0.11	−0.11	0.06	0.24	0.24	0.00	−0.38	−0.03	−0.04
Plant height	0.03	−0.10	**0.61** ^**a**^	−0.11	0.32	0.11	0.14	−0.11	0.29	0.17	0.16	−0.02	−0.12	0.09	0.32
Plant breadth	0.06	−0.03	**0.74** ^**a**^	0.26	−0.11	0.10	0.08	0.28	−0.15	−0.35	0.06	0.12	−0.04	0.01	−0.07
Canopy density	0.20	0.01	**0.86** ^**a**^	0.06	0.11	−0.04	0.07	0.04	−0.02	0.10	−0.02	0.12	−0.03	−0.09	0.07
Plant branching	0.20	0.15	**0.81** ^**a**^	0.29	−0.03	−0.03	−0.05	−0.10	0.01	0.11	−0.03	0.13	−0.07	0.12	−0.06
Stem diameter	0.06	0.03	**0.82** ^**a**^	0.00	−0.17	0.08	0.03	−0.03	0.04	0.05	−0.11	−0.19	0.11	−0.10	0.07
Main stem bark cracks	0.09	0.02	0.53	−0.02	0.18	0.01	0.01	−0.20	−0.07	−0.03	0.39	0.14	−0.24	−0.15	−0.46
Leaf density	0.04	−0.28	**0.73** ^**a**^	−0.09	0.00	0.03	−0.02	−0.09	0.05	−0.03	0.10	0.11	0.33	0.11	−0.15
Young leaf shape	0.34	0.26	0.03	−0.10	−0.05	−0.46	0.34	0.04	0.37	0.03	−0.16	−0.34	−0.18	0.22	−0.08
Young leaf length	0.44	0.13	0.24	−0.12	−0.06	0.16	**0.64** ^**a**^	0.25	−0.01	0.32	−0.06	0.06	−0.22	−0.02	−0.06
Young leaf width	0.38	0.07	0.24	−0.07	−0.15	0.09	**0.65** ^**a**^	0.33	0.17	0.15	−0.01	−0.24	−0.13	0.05	−0.08
Young leaf apex shape	0.18	0.10	0.18	−0.03	−0.07	0.03	0.06	0.07	−0.03	0.12	−0.03	**0.87** ^**a**^	−0.01	−0.08	0.03
Young leaf base shape	−0.12	0.09	0.05	0.03	0.11	−0.15	0.02	0.00	**0.88** ^**a**^	0.08	0.06	−0.05	0.00	−0.04	0.00
Young leaf color	−0.18	**0.87** ^**a**^	0.07	0.05	−0.06	−0.10	0.14	−0.18	0.07	−0.13	0.15	−0.10	0.07	0.01	0.15
Young leaf vein color	−0.18	**0.78** ^**a**^	−0.05	−0.26	0.11	−0.18	0.24	0.14	−0.06	0.12	0.04	0.05	0.03	−0.11	0.03
Young leaf pubescence	0.16	**0.73** ^**a**^	−0.03	−0.19	0.02	−0.18	−0.07	0.03	0.11	0.28	−0.17	0.04	−0.08	0.12	−0.26
Young leaf thickness	0.00	−0.42	0.05	0.48	−0.17	0.13	−0.07	−0.30	0.46	−0.18	0.11	−0.06	−0.13	0.08	−0.13
Mature leaf shape	0.14	0.05	0.08	−0.03	−0.02	**−0.65** ^**a**^	0.20	0.20	−0.09	−0.20	−0.40	0.28	0.08	−0.06	−0.05
Mature leaf length	0.08	0.36	0.07	−0.07	0.00	0.02	**0.86** ^**a**^	0.07	−0.08	0.06	−0.03	0.06	0.09	0.12	0.05
Mature leaf width	−0.07	0.03	−0.05	0.21	−0.04	−0.08	**0.84** ^**a**^	−0.17	0.04	−0.11	−0.10	0.09	0.15	0.04	0.11
Mature leaf apex shape	0.38	0.21	0.00	−0.15	−0.03	−0.17	0.28	0.05	−0.06	0.06	0.02	0.40	−0.23	−0.03	0.56
Mature leaf color	−0.24	**0.86** ^**a**^	0.10	0.03	−0.05	−0.06	0.21	−0.11	0.04	−0.16	0.08	−0.06	0.04	−0.02	0.18
Mature leaf vein color	−0.18	**0.69** ^**a**^	−0.07	−0.37	0.02	−0.15	0.29	−0.01	−0.03	0.09	0.04	0.23	0.08	−0.07	0.01
Mature leaf pubescence	0.04	**0.74** ^**a**^	−0.19	−0.26	−0.09	0.00	−0.09	−0.02	0.01	−0.05	0.02	0.13	0.06	0.11	−0.13
Mature leaf thickness	−0.03	0.06	−0.01	0.25	−0.38	0.09	−0.04	−0.47	0.52	−0.12	0.17	−0.04	0.03	−0.20	0.24
Inflorescence diameter	−0.22	−0.29	0.18	0.51	0.08	0.41	0.17	−0.39	0.16	0.07	0.12	0.06	0.09	0.19	−0.01
Number of flowers in inflorescence	−0.46	−0.38	0.02	0.50	0.12	0.26	−0.13	−0.40	−0.06	−0.01	0.16	0.12	0.10	0.04	−0.05
Peduncle length	**0.61** ^**a**^	0.10	0.08	0.03	0.18	−0.11	−0.05	0.33	−0.39	0.22	0.13	−0.04	0.16	−0.18	0.10
Peduncle diameter	0.37	−0.15	−0.04	0.37	0.00	0.01	0.04	0.11	−0.39	0.23	0.43	−0.22	−0.06	−0.19	0.19
Pedicel length	0.20	−0.32	−0.17	0.11	0.04	0.13	0.06	**0.78** ^**a**^	−0.06	−0.05	0.01	0.12	0.18	0.16	0.12
Pedicel diameter	0.11	−0.12	−0.01	0.27	−0.35	**0.63** ^**a**^	−0.01	−0.12	0.03	−0.15	0.22	−0.14	−0.33	−0.02	−0.06
Flower diameter	0.22	−0.14	0.19	**0.72** ^**a**^	0.11	0.13	0.01	0.08	0.13	0.06	−0.21	−0.15	−0.01	0.17	−0.06
Petal length	0.14	−0.11	0.11	**0.71** ^**a**^	−0.14	0.03	−0.06	−0.18	0.17	0.35	−0.10	−0.05	−0.01	−0.18	0.20
Petal width	0.10	−0.01	0.11	0.60	−0.04	**0.63** ^**a**^	−0.08	0.24	−0.03	0.12	−0.08	0.01	−0.04	−0.18	−0.07
Petal color	0.24	0.07	0.29	0.19	0.04	0.19	0.03	0.24	−0.06	−0.09	−0.04	−0.16	−0.04	**0.70** ^**a**^	0.16
Corona lobe length	−0.04	−0.28	−0.01	**0.79** ^**a**^	−0.02	0.19	0.09	0.06	−0.14	−0.04	−0.01	0.10	−0.01	−0.02	−0.05
Stigmatic disc width	−0.03	−0.20	−0.15	0.24	0.02	−0.03	0.14	0.04	−0.12	0.12	**−0.78** ^**a**^	−0.02	−0.04	−0.05	0.03
Sepal length	0.43	−0.27	0.10	0.22	−0.15	**0.66** ^**a**^	0.06	0.18	−0.12	−0.21	−0.09	−0.04	−0.14	0.07	−0.07
Sepal width	0.08	−0.22	0.04	0.15	0.14	**0.79** ^**a**^	0.15	0.04	−0.16	−0.01	−0.19	0.24	0.04	0.05	0.01
Fruit density	0.10	−0.01	0.19	0.26	0.12	0.00	0.09	0.01	−0.02	**0.74** ^**a**^	−0.07	0.15	0.09	0.13	0.02
Fruit length	**0.91** ^**a**^	−0.07	0.21	−0.02	0.03	0.14	−0.03	−0.03	−0.14	0.10	−0.03	0.04	−0.04	0.01	0.00
Fruit width	**0.78** ^**a**^	−0.34	0.16	0.20	−0.09	0.13	0.05	0.06	0.01	−0.17	0.03	0.09	−0.04	0.20	0.08
Fruit thickness	**0.68** ^**a**^	−0.39	0.06	0.18	−0.18	0.11	0.00	−0.04	−0.13	−0.23	0.12	0.23	−0.04	0.14	0.09
Fruit stalk length	−0.11	0.14	0.01	−0.03	0.16	−0.14	0.12	0.11	−0.03	0.08	0.02	−0.02	**0.84** ^**a**^	−0.15	−0.04
Fruit stalk diameter	**0.63** ^**a**^	0.08	−0.02	0.04	−0.27	−0.17	0.20	0.26	−0.05	0.06	0.12	0.27	0.25	−0.17	0.08
Fruit weight	**0.88** ^**a**^	−0.13	0.18	0.06	0.04	0.03	0.14	0.03	0.15	0.13	−0.05	−0.05	−0.19	0.04	−0.06
Fruit skin color	−0.54	0.52	−0.05	0.17	−0.15	0.06	−0.09	−0.38	0.02	−0.23	0.29	−0.08	0.05	−0.03	0.16
Seed length	−0.20	−0.20	0.01	0.14	**0.85** ^**a**^	−0.03	−0.04	0.02	0.13	0.13	0.02	0.11	0.08	0.14	−0.01
Seed width	0.27	0.16	0.13	−0.04	**0.68** ^**a**^	−0.12	0.11	0.04	0.19	0.27	−0.16	−0.28	−0.12	0.01	0.18
Seed thickness	0.42	0.05	0.02	−0.16	−0.36	−0.16	0.37	0.45	−0.17	−0.17	−0.17	0.08	0.22	0.09	−0.18
100-seed weight	−0.09	−0.03	−0.05	−0.04	**0.86** ^**a**^	−0.01	−0.14	0.02	−0.13	−0.17	0.12	−0.05	0.10	−0.10	−0.13
Seed hair length (fibers)	0.16	0.38	0.15	−0.31	0.58	0.17	0.01	−0.11	−0.18	0.17	−0.32	−0.03	0.21	0.16	0.05
*Eigenvalue*	*9.25*	*7.70*	*4.94*	*3.95*	*3.03*	*2.88*	*2.38*	*2.16*	*1.79*	*1.54*	*1.37*	*1.24*	*1.12*	*1.03*	*1.00*
*Component degree of significance*	**	**	**	**	**	**	**	*	*	*	*	*	*	*	*ns*
*Variance (%)*	*17.45*	*14.53*	*9.32*	*7.46*	*5.71*	*5.44*	*4.49*	*4.08*	*3.37*	*2.90*	*2.59*	*2.33*	*2.10*	*1.94*	*1.89*
*∑ variance (%)*	*17.45*	*31.98*	*41.30*	*48.76*	*54.47*	*59.91*	*64.40*	*68.48*	*71.85*	*74.75*	*77.33*	*79.67*	*81.77*	*83.72*	*85.60*

^a^Bold values indicate the characteristics that most influence each PC (Eigenvalues are significant ≥ 0.61). Component degree of significance: *p < 0.05, **p < 0.01, *ns* not significant.

The PCA results provide valuable insights into the morphological variation of *C. procera*, a neglected medicinal plant.

PC1 explains 17.45% of the total variation, with the highest loadings observed for fruit-related traits, such as fruit length (0.91), fruit weight (0.88), fruit width (0.78), fruit thickness (0.68), fruit stalk diameter (0.63), and peduncle length (0.61). This suggests that these fruit-related attributes are the most influential factors in explaining the morphological variation in *C. procera*. These traits are likely to be important for ecological and evolutionary studies of the plant, as they may affect seed dispersal, reproductive success, and adaptability to different environments.

PC2, which accounts for 14.53% of the variation, is predominantly influenced by leaf-related characteristics, including young leaf color (0.87), mature leaf color (0.86), young leaf vein color (0.78), mature leaf pubescence (0.74), young leaf pubescence (0.73), and mature leaf vein color (0.69). This indicates that leaf morphology, particularly color and pubescence, is a significant contributor to the morphological diversity observed in the species. These traits may be associated with environmental factors such as light intensity and water availability, which could influence the plant’s physiological processes and survival strategies.

PC3 explains 9.32% of the total variation, with the most significant contributions from canopy density (0.86), stem diameter (0.82), plant branching (0.81), plant breadth (0.74), leaf density (0.73), and plant height (0.61). These structural traits reflect the overall growth habit and architecture of the plant, which are essential for its competitive ability in various habitats. The positive correlations between these variables suggest that larger, denser plants with more branching may have a greater competitive advantage in resource acquisition, particularly in environments with limited light or space.

In summary, the relatively low cumulative variance explained by these three components (41.3%) indicates that additional factors, potentially including environmental influences, contribute substantially to the overall phenotypic variation in this species. The observed trait associations in each component may reflect underlying genetic linkages or developmental constraints that shape morphological integration in *C. procera*. The strong loading of fruit dimensions on PC1 suggests these traits could serve as reliable indicators for morphological characterization, while the leaf traits dominating PC2 may be particularly useful for distinguishing developmental stages or accessions. The architectural traits in PC3 might relate to plant ecological strategies and resource allocation patterns. These findings provide a quantitative framework for understanding the multivariate relationships between morphological traits in this understudied medicinal plant species.

Previous studies conducted on *C. procera* in Brazil [69], Benin [2], and Pakistan [70] have utilized PCA for data evaluation. However, since these studies did not incorporate morphological traits, the PCA results obtained in our study were analyzed independently among themselves. This approach provides a unique analytical framework by enabling an unbiased interpretation of morphological variation within the species.

A biplot enables the simultaneous visualization of two or more components, facilitating a more straightforward understanding of the relationships and structure within the data [71]. In a biplot, each observation in the dataset is represented as a point on a plane defined by specific components. Each axis corresponds to one of the principal components derived from the dataset. Alongside the observations, arrows representing the variables are also displayed. These arrows indicate the relationships between variables and the contribution of each variable to the principal components. Biplot analysis is typically applied to quantitative data, as the linear relationships and variance between the variables can be meaningfully depicted through such visualizations [72]. This method is particularly useful in presenting the effects of multiple variables in a dataset in a more simplified and interpretable manner, especially when dealing with a large number of variables [73].

**Fig 7:**
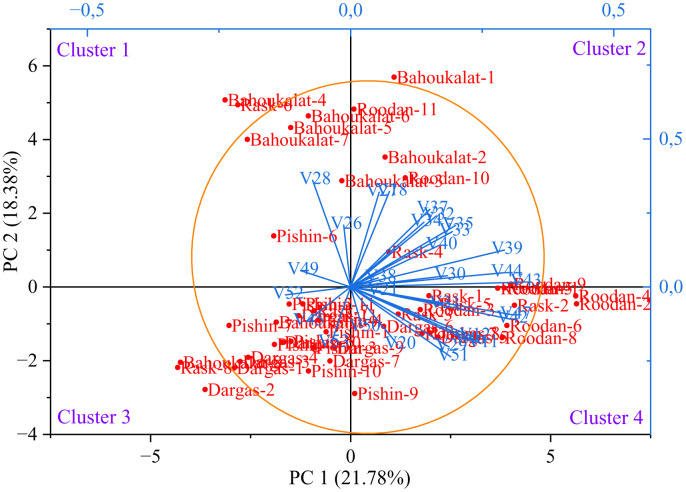
Biplot for the studied *Calotropis procera* accessions and quantitative variables based on PC1/PC2 of morphological traits. For abbreviations, please see Table 1. [Accessions, – Quantitative variables, O = 95% Confidence ellipse].

Biplot for the studied *C. procera* accessions and quantitative variables based on PC1/PC2 of morphological traits is visualized in Fig 7. The two-dimensional biplot analysis revealed a clear distribution of both variables and accessions across four distinct clusters, with principal component 1 (PC1) accounting for 21.78% of the total variation and principal component 2 (PC2) explaining 18.38%. Collectively, the first two principal components represented 40.16% of the total variability, indicating a moderate but meaningful differentiation among the studied accessions of *C. procera* based on morphological traits.

Cluster 1 included the accessions ‘Bahoukalat-3’, ‘Bahoukalat-4’, ‘Bahoukalat-5’, ‘Bahoukalat-6’, ‘Bahoukalat-7’, ‘Pishin-6’, and ‘Rask-6’, which were primarily associated with mature leaf thickness, the number of flowers in the inflorescence, and seed length. The grouping of these variables suggests that these accessions exhibit greater floral production potential and robust leaf development, which may have ecological and agronomic implications in arid and semi-arid environments where *C. procera* thrives.

Cluster 2 comprised ‘Bahoukalat-1’, ‘Bahoukalat-2’, ‘Rask-4’, ‘Roodan-9’, ‘Roodan-10’, and ‘Roodan-11’, which were linked to young leaf thickness, inflorescence diameter, peduncle diameter, pedicel diameter, flower diameter, petal length, petal width, corona lobe length, stigmatic disc width, sepal length, sepal width, fruit width, and fruit thickness. The presence of these morphological traits in this cluster suggests that these accessions may exhibit larger floral structures and more robust reproductive traits, which could influence pollination success and seed dispersal mechanisms.

Cluster 3 included ‘Bahoukalat-8’, ‘Bahoukalat-9’, ‘Dargas-1’, ‘Dargas-2’, ‘Dargas-3’, ‘Dargas-4’, ‘Dargas-7’, ‘Dargas-9’, ‘Dargas-10’, ‘Dargas-11’, ‘Pishin-1’, ‘Pishin-2’, ‘Pishin-3’, ‘Pishin-4’, ‘Pishin-5’, ‘Pishin-7’, ‘Pishin-8’, ‘Pishin-10’, ‘Pishin-11’, ‘Rask-7’, and ‘Rask-8’, which were mainly associated with fruit stalk length, 100-seed weight, and seed hair length (fibers). The morphological characteristics of these accessions suggest a potential adaptation to seed dispersal via wind due to the presence of longer seed hairs, which enhance buoyancy. Additionally, the higher 100-seed weight observed in these accessions may indicate better seed viability and germination potential, factors that could be relevant for conservation and propagation strategies.

Cluster 4 included ‘Dargas-5’, ‘Dargas-6’, ‘Dargas-8’, ‘Pishin-9’, ‘Rask-1’, ‘Rask-2’, ‘Rask-3’, ‘Rask-5’, ‘Roodan-1’, ‘Roodan-2’, ‘Roodan-3’, ‘Roodan-4’, ‘Roodan-5’, ‘Roodan-6’, ‘Roodan-7’ and ‘Roodan-8’, which were associated with young leaf length, young leaf width, mature leaf length, mature leaf width, peduncle length, pedicel length, fruit length, fruit stalk diameter, fruit weight, seed width, and seed thickness. The predominance of leaf-related traits in this cluster suggests that these accessions may have superior vegetative growth, which could contribute to greater photosynthetic efficiency and biomass production. Additionally, the larger fruit dimensions observed in this cluster may indicate a higher reproductive output, potentially influencing seed dispersal and plant establishment.

The fact that the accessions ‘Bahoukalat-1’, ‘Bahoukalat-4’, ‘Bahoukalat-8’, ‘Dargas-2’, ‘Rask-6’, ‘Rask-8’, ‘Roodan-2’, and ‘Roodan-4’ fall outside the 95% confidence ellipse indicates that these accessions exhibit distinct morphological traits compared to the majority of the population under analysis. The confidence ellipse represents a region that reflects the expected distribution of 95% of the data points. When accessions lie outside this region, it implies that they possess unique or extraordinary characteristics that set them apart from others [74,75,76]. These accessions may represent genetically distinct accessions that have adapted to different environmental conditions or undergone different evolutionary pressures.

The observed clustering patterns underscore significant morphological diversity among *C. procera* accessions, likely driven by local ecological adaptations or genetic divergence. The differential distribution of traits across clusters further emphasizes the species’ phenotypic plasticity, which may contribute to its success in varied environments. However, the moderate cumulative variance explained by the first two PCs suggests that additional factors, possibly including environmental influences or genetic variations, may further shape the morphological differentiation in this species. These findings provide a valuable foundation for future studies on genetic resource conservation, breeding programs, and the utilization of this medicinally important species. The identification of trait-specific clusters can facilitate the selection of superior accessions for targeted breeding efforts, ensuring the sustainable use of *C. procera* in both ecological and pharmaceutical applications.

Biplot analysis has been employed in studies conducted on *C. procera* in Brazil [69], Benin [2], Saudi Arabia and Egypt [77], Iran [78], and Pakistan [70,58]. However, since these previous studies did not incorporate morphological variables, the findings obtained in our research were evaluated independently and exclusively among themselves. This approach provides our survey with a unique analytical perspective and allows for a more accurate interpretation of the internal structure of morphological variation.

**Fig 8:**
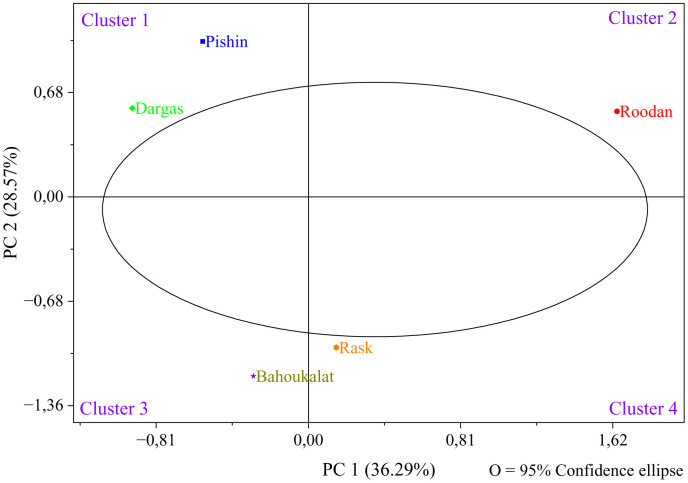
Population analysis showing the score plot of the studied *Calotropis procera* accessions grouped according to their regions of collection based on PC1 and PC2.

Population analysis (Fig 8) showed that PC1 explained 36.29% of the total variance, while PC2 accounted for 28.57%, with the first two components jointly representing 64.86% of the overall variation. As summarized in Table 5, the first four principal components exhibited eigenvalues of 19.23, 15.14, 12.56, and 6.07, explaining 36.29%, 28.57%, 23.69%, and 11.45% of the variance, respectively, and together accounting for 100.00% of the total variation. The relatively high cumulative contribution of PC1 and PC2 supports a robust visual discrimination of accessions according to their regions of collection in the PC1–PC2 ordination space. Consistent with this pattern, the regions were separated into four distinct clusters: Cluster 1 comprised predominantly the Dargas and Pishin accessions, Cluster 2 included the Roodan accessions, Cluster 3 contained the Bahoukalat accessions, and Cluster 4 corresponded mainly to the Rask accessions. This distribution suggests that certain regions exhibit closer morphological similarity (e.g., Dargas–Pishin), whereas others show more pronounced divergence (e.g., Roodan, Bahoukalat, and Rask). The observed clustering structure likely reflects the combined effects of environmental factors (e.g., microclimate, edaphic conditions, and habitat heterogeneity) and inherent gene flow and phenotypic plasticity in natural populations [79].

**Table 5:** Eigenvalues derived from the correlation matrix associated with the population analysis of *Calotropis procera* accessions.

PC No.	Eigenvalue	Percentage of Variance	Cumulative	Component degree of significance
1	19.23	36.29%	36.29%	**
2	15.14	28.57%	64.86%	**
3	12.56	23.69%	88.55%	**
4	6.07	11.45%	100.00%	**

*PC* principal component. Component significance: **p < 0.01.

### 3.5. Heat map analysis (HMA)

Heat map analysis, utilizing Ward’s method and Euclidean distance coefficients, is an effective approach for visualizing and analyzing relationships in complex datasets [80]. Ward’s method is a hierarchical clustering technique that merges clusters by minimizing the increase in within-cluster variance, leading to compact, well-separated clusters [81]. This method is particularly useful for identifying natural groupings within data. Euclidean distance coefficients measure the dissimilarity between observations. These distances are used to create a distance matrix, which is then displayed as a heat map [82]. The color intensity on the heat map reflects the degree of similarity or dissimilarity between data points, making it easy to spot patterns, correlations, or outliers. This analysis is especially suitable for quantitative data, as it visually demonstrates the underlying structure of the dataset, highlights clusters, and reveals potential relationships between variables [83]. Heat map analysis, combined with Ward’s method and Euclidean distance, is invaluable for exploring large datasets and providing insights for further statistical analysis or decision-making.

**Fig 9:**
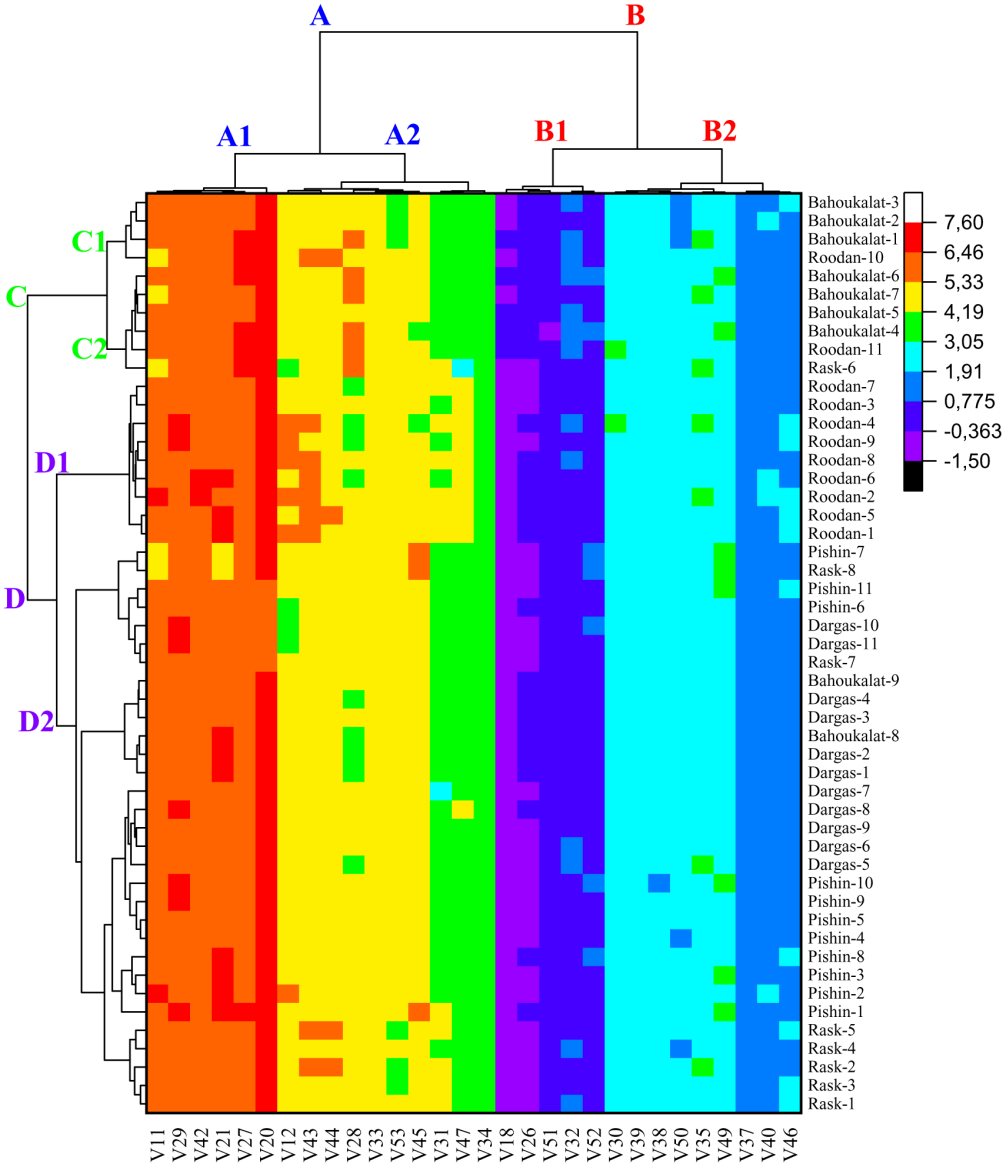
Visualization of clustering patterns of *Calotropis procera* accessions and variables based on morphological characterizations using a heat map. For abbreviations, please see Table 1.

The morphological characterization of *C. procera*, based on clustering analysis, demonstrates a structured grouping of both variables and accessions. The heat map visualization indicates that the variables are initially divided into two major groups, A and B, which are further subdivided into A1, A2, B1, and B2 subgroups. Similarly, the accessions are classified into two main clusters, C and D, which are further divided into C1, C2, D1, and D2 subgroups. The association between accessions and variable clusters provides insights into the morphological relationships within this species (Fig 9).

In subgroup A1, which includes young leaf length, mature leaf length, mature leaf width, inflorescence diameter, peduncle length, and fruit length, the accessions associated with this cluster predominantly belong to the C1 subgroup. Specifically, accessions such as ‘Roodan-10’, ‘Bahoukalat-1’, ‘Bahoukalat-2’, and ‘Bahoukalat-3’ exhibit morphological traits aligned with these variables, suggesting a potential relationship in terms of leaf size, inflorescence structure, and fruit elongation.

Conversely, subgroup A2, which encompasses young leaf width, number of flowers in inflorescence, pedicel length, flower diameter, petal length, fruit width, fruit thickness, fruit stalk length, fruit weight, and seed hair length (fibers), is closely associated with accessions from the C2 subgroup. Accessions such as ‘Rask-6’, ‘Roodan-11’, ‘Bahoukalat-4’, ‘Bahoukalat-5’, ‘Bahoukalat-6’, and ‘Bahoukalat-7’ exhibit morphological characteristics that correspond to floral traits, fruit dimensions, and seed fiber length, indicating a distinct grouping based on reproductive structures and fruit morphology.

Within group B, subgroup B1, which includes young leaf thickness, mature leaf thickness, pedicel diameter, seed thickness, and 100-seed weight, is predominantly associated with accessions from the D1 subgroup. Accessions such as ‘Roodan-1’ to ‘Roodan-9’ display traits linked to leaf and seed thickness, suggesting a morphological pattern that emphasizes structural robustness and seed mass.

Subgroup B2, which comprises peduncle diameter, petal width, corona lobe length, stigmatic disc width, sepal length, sepal width, fruit stalk diameter, seed length, and seed width, exhibits strong associations with accessions from the D2 subgroup. This group includes a diverse set of accessions, such as ‘Rask-1’ to ‘Rask-8’, ‘Pishin-1’ to ‘Pishin-11’, ‘Dargas-1’ to ‘Dargas-11’, ‘Bahoukalat-8’, and ‘Bahoukalat-9’. The traits in this group are primarily related to floral dimensions, reproductive structures, and seed morphology, suggesting that these accessions share common characteristics associated with reproductive fitness and seed dispersal mechanisms.

The structured clustering of variables and accessions highlights the morphological variability within *C. procera*, indicating that distinct accession groups exhibit specific trait associations. This classification provides valuable insights for future studies on the genetic diversity, ecological adaptation, and potential medicinal applications of this neglected species.

Studies on *C. procera* in Pakistan [70,58] employed HMA. However, due to the previous researchers’ use of different variable parameters, our findings were subjected to an independent evaluation within our own dataset.

## 4. Conclusions

This study represents comprehensive research systematically evaluating the morphological diversity of *C. procera* accessions collected from the Sistan-va-Baluchestan province of Iran. Among the 50 accessions examined in this study, significant morphological variations were observed, particularly in fruit, leaf, and reproductive characteristics. These findings highlight the species’ potential for adaptation to arid conditions and its phenotypic plasticity.

Multiple regression analyses revealed relationships between morphological traits. The most notable finding was the inverse correlation between flower yield and fruit development. This result provides important insights into the species’ reproductive strategy and resource allocation. PCA identified fruit size and leaf characteristics as key determinants of morphological variation. Specific accessions, such as ‘Roodan-10’ and ‘Pishin-8’, exhibited superior traits that could be valuable for breeding programs.

HMA revealed patterns of local adaptation among the accessions. For instance, accessions from the Bahoukalat region exhibited enhanced floral traits, while samples from Roodan demonstrated superior fruit characteristics. These findings provide important implications for selecting suitable genetic material for medicinal or ecological applications.

This study significantly contributes to our understanding of the morphological diversity of *C. procera*; however, it also highlights the need for molecular analyses to fully elucidate the genetic basis of the observed variation. Future research integrating the morphological data presented here with genomic approaches will facilitate the development of comprehensive strategies for the conservation and utilization of this ecologically and pharmacologically important species.

In conclusion, this research provides a foundation for further studies on *C. procera* and offers both methodological insights and practical data for the sustainable management of the species’ genetic resources. The detailed morphological characterization presented here will ease the identification of superior accessions for targeted applications and contribute to a better understanding of the biology of this underexplored medicinal plant.

## Supporting information

S1 Table Simple correlations between the quantitative morphological variables utilized in the studied *Calotropis procera* accessions. (DOCX)

S1 File Inclusivity-in-global-research-questionnaire. (DOCX)
