# Supplementary material for: Morphological characterization of Calotropis procera (Aiton) W.T. Aiton, a neglected medicinal plant
Source: PLoS One. 2026 Feb 10;21(2):e0341425. doi: 10.1371/journal.pone.0341425 (PMC12890142; doi:10.1371/journal.pone.0341425)
Supplement: S1 Table — (DOCX) [file pone.0341425.s001.docx]

**S1 Table.** Simple correlations between the quantitative morphological variables utilized in studied *Calotropis procera* accessions.

| Variables |  | V11 | V12 | V18 | V20 | V21 | V26 | V27 | V28 | V29 | V30 | V31 | V32 | V33 | V34 | V35 |
| --- | --- | --- | --- | --- | --- | --- | --- | --- | --- | --- | --- | --- | --- | --- | --- | --- |
| V11 |  | 1.00 |  |  |  |  |  |  |  |  |  |  |  |  |  |  |
| V12 |  | **0.86**** | 1.00 |  |  |  |  |  |  |  |  |  |  |  |  |  |
| V18 |  | -0.22 | -0.06 | 1.00 |  |  |  |  |  |  |  |  |  |  |  |  |
| V20 |  | **0.67**** | **0.63**** | **-0.36*** | 1.00 |  |  |  |  |  |  |  |  |  |  |  |
| V21 |  | **0.32*** | **0.33*** | 0.05 | **0.73**** | 1.00 |  |  |  |  |  |  |  |  |  |  |
| V26 |  | -0.22 | -0.06 | **0.52**** | -0.13 | 0.16 | 1.00 |  |  |  |  |  |  |  |  |  |
| V27 |  | -0.07 | -0.07 | **0.55**** | 0.02 | 0.24 | **0.32*** | 1.00 |  |  |  |  |  |  |  |  |
| V28 |  | **-0.47**** | **-0.47**** | **0.51**** | **-0.33*** | 0.04 | 0.27 | **0.77**** | 1.00 |  |  |  |  |  |  |  |
| V29 |  | **0.36*** | 0.23 | **-0.44**** | 0.12 | -0.17 | **-0.33*** | **-0.32*** | **-0.38**** | 1.00 |  |  |  |  |  |  |
| V30 |  | 0.16 | 0.13 | 0.06 | 0.00 | 0.02 | -0.03 | 0.05 | 0.08 | **0.57**** | 1.00 |  |  |  |  |  |
| V31 |  | 0.17 | 0.21 | -0.10 | 0.02 | 0.00 | **-0.39**** | -0.11 | -0.17 | **0.30*** | 0.23 | 1.00 |  |  |  |  |
| V32 |  | 0.08 | 0.11 | **0.52**** | -0.15 | -0.06 | **0.31*** | **0.40**** | **0.29*** | -0.20 | 0.23 | 0.00 | 1.00 |  |  |  |
| V33 |  | 0.13 | 0.17 | **0.41**** | -0.06 | 0.09 | 0.06 | **0.46**** | **0.29*** | 0.09 | 0.22 | 0.18 | 0.20 | 1.00 |  |  |
| V34 |  | 0.04 | 0.03 | **0.44**** | -0.16 | 0.08 | **0.40**** | **0.44**** | **0.33*** | 0.02 | **0.31*** | -0.05 | 0.19 | **0.61**** | 1.00 |  |
| V35 |  | 0.17 | 0.13 | 0.28 | -0.09 | -0.05 | 0.09 | **0.39**** | **0.30*** | 0.08 | **0.29*** | **0.32*** | **0.50**** | **0.56**** | **0.54**** | 1.00 |
| V37 |  | -0.05 | -0.05 | **0.40**** | -0.05 | 0.20 | 0.12 | **0.53**** | **0.55**** | 0.03 | **0.34*** | 0.23 | **0.39**** | **0.52**** | **0.49**** | **0.57**** |
| V38 |  | 0.07 | 0.05 | 0.07 | 0.04 | 0.17 | -0.15 | 0.02 | 0.04 | 0.01 | -0.08 | 0.14 | -0.08 | 0.21 | 0.20 | 0.20 |
| V39 |  | **0.29*** | 0.27 | **0.29*** | 0.00 | 0.01 | -0.03 | 0.26 | 0.08 | 0.20 | **0.28*** | **0.38**** | **0.62**** | **0.40**** | 0.14 | **0.61**** |
| V40 |  | 0.25 | 0.10 | 0.10 | 0.10 | 0.13 | -0.09 | **0.44**** | **0.32*** | 0.04 | 0.07 | **0.28*** | **0.38**** | **0.28*** | 0.12 | **0.54**** |
| V42 |  | **0.50**** | **0.36**** | -0.06 | 0.06 | -0.13 | -0.13 | -0.09 | **-0.34*** | **0.61**** | **0.36**** | 0.17 | 0.15 | 0.24 | 0.20 | 0.23 |
| V43 |  | **0.34*** | **0.35*** | 0.27 | 0.00 | 0.01 | 0.00 | 0.14 | -0.10 | **0.36*** | **0.32*** | **0.41**** | **0.30*** | **0.41**** | 0.19 | 0.25 |
| V44 |  | 0.20 | 0.16 | 0.27 | -0.07 | 0.00 | 0.02 | 0.14 | 0.01 | **0.30*** | **0.35*** | **0.36**** | **0.33*** | **0.29*** | 0.13 | 0.18 |
| V45 |  | -0.12 | -0.08 | -0.23 | 0.19 | 0.17 | -0.11 | -0.04 | -0.02 | 0.23 | -0.06 | 0.14 | **-0.42**** | -0.14 | -0.07 | -0.09 |
| V46 |  | **0.43**** | **0.39**** | -0.15 | 0.28 | 0.16 | -0.02 | **-0.35*** | **-0.44**** | **0.51**** | **0.28*** | **0.33*** | -0.05 | 0.01 | 0.15 | 0.04 |
| V47 |  | **0.59**** | **0.54**** | 0.15 | 0.13 | -0.02 | -0.04 | -0.04 | **-0.40**** | **0.48**** | **0.28*** | 0.16 | 0.17 | **0.32*** | 0.26 | 0.16 |
| V49 |  | -0.18 | -0.24 | 0.04 | -0.10 | -0.05 | **-0.31*** | **0.33*** | **0.35*** | 0.01 | -0.07 | 0.09 | **-0.31*** | 0.22 | 0.00 | -0.01 |
| V50 |  | **0.28*** | 0.25 | -0.17 | 0.14 | 0.05 | -0.20 | -0.12 | -0.25 | 0.27 | 0.08 | 0.00 | -0.27 | 0.19 | 0.06 | -0.04 |
| V51 |  | **0.48**** | **0.51**** | -0.25 | **0.40**** | 0.18 | **-0.33*** | **-0.38**** | **-0.55**** | **0.35*** | -0.04 | **0.44**** | -0.08 | -0.01 | -0.22 | -0.04 |
| V52 |  | -0.27 | -0.27 | -0.15 | -0.15 | -0.14 | **-0.32*** | -0.06 | 0.17 | 0.10 | 0.00 | 0.04 | **-0.29*** | -0.04 | -0.24 | -0.03 |
| V53 |  | 0.18 | 0.03 | **-0.52**** | 0.26 | -0.06 | **-0.39**** | -0.13 | -0.23 | 0.23 | -0.17 | -0.10 | **-0.35*** | -0.05 | -0.21 | -0.06 |

**Supplementary T1.** Continued.

| Variables | V37 | V38 | V39 | V40 | V42 | V43 | V44 | V45 | V46 | V47 | V49 | V50 | V51 | V52 | V53 |
| --- | --- | --- | --- | --- | --- | --- | --- | --- | --- | --- | --- | --- | --- | --- | --- |
| V37 | 1.00 |  |  |  |  |  |  |  |  |  |  |  |  |  |  |
| V38 | **0.31*** | 1.00 |  |  |  |  |  |  |  |  |  |  |  |  |  |
| V39 | **0.40**** | 0.12 | 1.00 |  |  |  |  |  |  |  |  |  |  |  |  |
| V40 | **0.37**** | 0.19 | **0.70**** | 1.00 |  |  |  |  |  |  |  |  |  |  |  |
| V42 | 0.03 | -0.02 | **0.49**** | 0.23 | 1.00 |  |  |  |  |  |  |  |  |  |  |
| V43 | 0.24 | 0.03 | **0.61**** | **0.28*** | **0.75**** | 1.00 |  |  |  |  |  |  |  |  |  |
| V44 | 0.27 | -0.01 | **0.57**** | 0.26 | **0.65**** | **0.93**** | 1.00 |  |  |  |  |  |  |  |  |
| V45 | -0.09 | 0.05 | **-0.30*** | -0.11 | -0.15 | -0.25 | -0.27 | 1.00 |  |  |  |  |  |  |  |
| V46 | 0.05 | -0.07 | 0.12 | -0.03 | **0.52**** | **0.44**** | **0.43**** | 0.18 | 1.00 |  |  |  |  |  |  |
| V47 | 0.04 | 0.04 | **0.46**** | 0.14 | **0.86**** | **0.75**** | **0.59**** | -0.21 | **0.48**** | 1.00 |  |  |  |  |  |
| V49 | 0.09 | 0.07 | -0.21 | 0.17 | -0.14 | -0.10 | -0.17 | 0.20 | **-0.32*** | -0.07 | 1.00 |  |  |  |  |
| V50 | -0.20 | 0.11 | -0.13 | -0.08 | 0.23 | 0.07 | -0.15 | 0.06 | -0.04 | **0.35*** | **0.47**** | 1.00 |  |  |  |
| V51 | -0.06 | 0.14 | 0.24 | -0.05 | **0.35*** | **0.37**** | **0.33*** | 0.22 | **0.60**** | **0.36*** | **-0.40**** | -0.16 | 1.00 |  |  |
| V52 | -0.01 | -0.09 | -0.17 | 0.05 | -0.08 | -0.18 | -0.20 | 0.20 | -0.25 | -0.14 | **0.66**** | **0.42**** | **-0.33*** | 1.00 |  |
| V53 | **-0.29*** | 0.13 | -0.12 | 0.16 | 0.27 | -0.09 | -0.20 | 0.25 | -0.07 | 0.10 | **0.33*** | **0.55**** | -0.03 | **0.44**** | 1.00 |

****p*<0.05**, *****p*<0.01**. For abbreviations, please see Table 1.
